# Biogenesis of the crystalloid organelle in *Plasmodium* involves microtubule-dependent vesicle transport and assembly

**DOI:** 10.1016/j.ijpara.2015.03.002

**Published:** 2015-07

**Authors:** Sadia Saeed, Annie Z. Tremp, Johannes T. Dessens

**Affiliations:** Pathogen Molecular Biology Department, Faculty of Infectious and Tropical Diseases, London School of Hygiene & Tropical Medicine, Keppel Street, London WC1E 7HT, United Kingdom

**Keywords:** Crystalloid, Cargo transport, LCCL protein, Transgenic parasite, *Plasmodium berghei*

## Abstract

Malaria parasites possess unique subcellular structures and organelles. One of these is the crystalloid, a multivesicular organelle that forms during the parasite’s development in vector mosquitoes. The formation and function of these organelles remain poorly understood. A family of six conserved and modular proteins named LCCL-lectin adhesive-like proteins (LAPs), which have essential roles in sporozoite transmission, localise to the crystalloids. In this study we analyse crystalloid formation using transgenic *Plasmodium berghei* parasites expressing GFP-tagged LAP3. We show that deletion of the LCCL domain from LAP3 causes retarded crystalloid development, while knockout of LAP3 prevents formation of the organelle. Our data reveal that the process of crystalloid formation involves active relocation of endoplasmic reticulum-derived vesicles to common assembly points via microtubule-dependent transport. Inhibition of microtubule-dependent cargo transport disrupts this process and replicates the LCCL domain deletion mutant phenotype in wildtype parasites. These findings provide the first clear insight into crystalloid biogenesis, demonstrating a fundamental role for the LAP family in this process, and identifying the crystalloid and its formation as potential targets for malaria transmission control.

## Highlights

• Crystalloid formation in *Plasmodium berghei* occurs during the early phase of ookinete development.
• Deletion of the LCCL domain from LAP3 causes delayed crystalloid formation.
• Knockout of LAP3 prevents crystalloid formation.
• Crystalloid biogenesis involves active vesicle transport and assembly.
• Crystalloid assembly is microtubule-dependent.

## Graphical abstract

**Figure:**
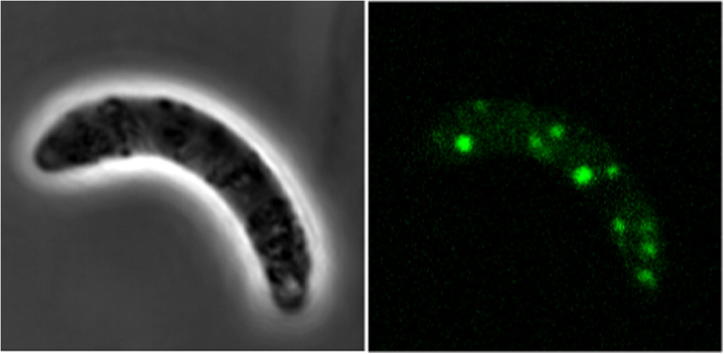

## 1 Introduction

Reducing parasite transmission by mosquitoes is an essential part of successful malaria control and eradication programmes. Malaria transmission starts with the uptake of the sexual stages (gametocytes) with the blood meal of a feeding vector mosquito, which initiates rapid gametogenesis and fertilisation. The resulting zygotes transform over a 16–24 h period into motile elongated stages called ookinetes, which cross the midgut epithelium of the insect and then round up and transform into oocysts. In the ensuing 2–3 weeks, the oocysts grow and differentiate to generate thousands of progeny sporozoites. After egress from the oocysts, the sporozoites invade and inhabit the salivary glands, and are transmitted to new hosts by mosquito bite to initiate new malaria infections.

*Plasmodium* crystalloids are transient parasite organelles that are uniquely found in ookinetes and young oocysts (Dessens et al., 2011). The organelles have been identified in human, monkey, rodent and bird malaria species, appearing in transmission electron microscopy (TEM) as clusters, 0.5–2.0 μm in diameter, of small spherical subunits. These subunits, 35–55 nm in diameter, have been shown in high-resolution TEM to be individually bound by a lipid bilayer, indicating that they constitute small vesicles (Garnham et al., 1962, 1969; Trefiak and Desser, 1973; Terzakis et al., 1976; Meis and Ponnudurai, 1987). In rodent malaria species, crystalloids are associated with larger vesicles containing hemozoin (also known as the malaria pigment, a product of haem detoxification in the food vacuoles) (Garnham et al., 1969; Sinden et al., 1985; Carter et al., 2008).

Thus far, the only parasite proteins found to localise to crystalloids are a family of six gametocyte-expressed proteins named LCCL-lectin adhesive-like proteins (LAPs) (Carter et al., 2008; Saeed et al., 2010, 2013). LAPs are highly conserved between *Plasmodium* spp. and possess a modular architecture comprised of multiple domains implicated in protein, lipid and carbohydrate binding (Claudianos et al., 2002; Delrieu et al., 2002; Pradel et al., 2004; Trueman et al., 2004). LAPs were named after the *Limulus* clotting factor C, cochlear protein 5b2, and lung gestation protein 1 (LCCL) module (Trexler et al., 2000), which is present in single or multiple copies in all but one family member. In addition, the LAPs possess an amino-terminal endoplasmic reticulum (ER) signal peptide. *Plasmodium* LAPs are predominantly expressed in female gametocytes and, following gametogenesis and fertilisation, they efficiently redistribute from the ER to the crystalloids during ookinete development and are subsequently carried over to the young oocyst with the organelles (Carter et al., 2008; Saeed et al., 2010, 2013). Based on available genome data, LAPs appear to be conserved across the Apicomplexa, albeit with some variation in the repertoire of LAP family members between genera (Claudianos et al., 2002; Dessens et al., 2004; Lavazec et al., 2009). The uniqueness, complexity and conservation of the LAP architectures strongly suggest that these proteins possess orthologous functions (Lavazec et al., 2009). By contrast, although some genera such as *Cryptosporidium* and *Cystoisospora* possess crystalloid-like structures, crystalloids appear not to be generally conserved in the Apicomplexa. A link between LAPs and crystalloids outside the genus *Plasmodium* is therefore not apparent. There is strong evidence that the *Plasmodium* LAPs are involved in sporozoite transmission: knockout of five of the family members in *Plasmodium berghei*, either as single or double knockouts, gives rise to arrested sporozoite development in the oocyst and subsequent failure of the parasite to be transmitted by mosquito bite (Claudianos et al., 2002; Raine et al., 2007; Carter et al., 2008; Ecker et al., 2008; Lavazec et al., 2009). In *Plasmodium falciparum* it has been shown that knockout of LAP1 (*Pf*CCp3) and LAP4 (*Pf*CCp2) results in loss of sporozoite transmission (Pradel et al., 2004). Several studies have furthermore shown that the LAPs interact with each other, and are interdependent for correct folding and stability (Pradel et al., 2006; Simon et al., 2009; Saeed et al., 2012), indicating that they operate as a protein complex.

Within several hours of fertilisation, spherical *Plasmodium* zygotes undergo DNA replication followed by meiotic division (Sinden et al., 1985; Janse et al., 1986). During meiosis, spindle microtubules form in the intact nucleus, which are organised from spindle pole plaques embedded in the nuclear membrane (Sinden et al., 1985). The apical complex, initially consisting of two polar rings, is formed under the zygote surface and goes on to form a protrusion. As zygote-to-ookinete transformation advances, this protrusion increases in size at the expense of the spherical progenitor zygote, ultimately forming the mature, banana-shaped ookinete, typically by 18–20 h post-fertilisation (Aikawa et al., 1984; Sinden et al., 1985). Intermediate stages (i.e. part spherical zygote, part elongated ookinete) are known as retorts. Concurrent with the formation of the apical protrusion, a unique cortical structure forms at the site where the protrusion extends from the zygote. This structure, known as the pellicle, is composed of the plasma membrane; an underlying double membrane structure called the inner membrane complex; and a cytoskeletal network of intermediate filaments termed the subpellicular network (Mann and Beckers, 2001; Morrissette and Sibley, 2002; Khater et al., 2004). Underlying the pellicle are subpellicular microtubules that originate at the polar rings and extend toward the posterior end of the ookinete (Aikawa et al., 1984; Sinden et al., 1985; Morrissette and Sibley, 2002). Besides subpellicular and spindle pole microtubules, cytoplasmic microtubules that appear to originate from at least two cytoplasmic centrioles have been observed in *Plasmodium* zygotes (Aikawa et al., 1984).

To date, virtually nothing is known about how crystalloids are formed. In this study, we used LAP3 in the rodent malaria parasite species *P. berghei* (PBANKA_020450) to carry out a detailed study of crystalloid formation. The results obtained provide unique new insight into the processes underlying crystalloid biogenesis, and identify a clear functional relationship between LAP expression, crystalloid formation and sporozoite transmission of malaria parasites. Our data also point to a prominent role of microtubules in crystalloid genesis. The biological significance of these findings with respect to LAP function in apicomplexan parasites is discussed.

## 2 Materials and methods

### 2.1 Animal use

All laboratory animal work undergoes regular ethical review by the London School of Hygiene & Tropical Medicine, UK, and was approved by the United Kingdom Home Office. Work was carried out in accordance with the United Kingdom Animals (Scientific Procedures) Act 1986 implementing European Directive 2010/63 for the protection of animals used for experimental purposes. Experiments were conducted in 6–8 week-old female CD1 mice, specific pathogen-free and maintained in filter cages. Animal welfare was assessed daily and animals were humanely killed upon reaching experimental or clinical endpoints. Mice were infected with parasites suspended in RPMI or PBS by i.p. injection, or by infected mosquito bite on anaesthetised animals. Parasitemia was monitored regularly by collecting a small volume of blood from a superficial tail vein. Drugs were administered by i.p. injection or where possible were supplied in drinking water. Parasitised blood was harvested by cardiac bleed under general anaesthesia without recovery.

### 2.2 Parasite maintenance, culture and transmission

*Plasmodium berghei* (ANKA clone 234) parasites were maintained as cryopreserved stabilates or by mechanical blood passage and regular mosquito transmission. To purify parasites for genomic DNA extraction, white blood cells were removed from parasitemic blood by passage through CF11 columns (Whatman, United Kingdom). Ookinete cultures were set up overnight from gametocytemic blood (Arai et al., 2001). Mosquito infection and transmission assays were as described using *Anopheles stephensi* (Dessens et al., 1999; Khater et al., 2004) and infected insects were maintained at 20 °C at approximately 70% relative humidity. Cell viability assays based on propidium iodide exclusion were carried out as described (Al-Khattaf et al., 2015). Briefly, cell viability was scored by fluorescence microscopy in the presence of 5 ml/L of propidium iodide and 1% Hoechst 33258. Ookinetes whose nucleus stained positive for both propidium iodide and Hoechst were scored as non-viable, whereas ookinetes whose nucleus only stained positive for Hoechst were scored as viable.

### 2.3 Generation and genomic analysis of transgenic parasite lines

Plasmid pLP-*Pb*LAP3/EFGP (Saeed et al., 2010) served as a template for inverse PCR using primers LAP3-KO-F (5′ATTCAAAAAGCTTAGGGGCCCTCAT3′) and LAP3-KO-R (5′CCTAAGCTTTTTGAATATATTAAAATGGTTGTAATAACCA3′). The amplified plasmid DNA was circularised via In-Fusion cloning (Takara Bio, Japan), resulting in the transfection construct pLP-*Pb*LAP3-KO, in which all but the first 21 codons of *P. berghei* LAP3 (*pblap3*) have been removed. The same was done with primers LAP3-LCCLKO-F (5′ACCATCATCCTTTATATTACTCAATACCAAATAGCTATTCA3′) and LAP3-LCCLKO-R2 (5′TATAAAGGATGATGGTTCATATATTCATTATCTATTATATTACATGA3′) to generate the transfection construct pLP-*Pb*LAP3/LCCL-KO, in which the entire LCCL domain, corresponding to amino acids 708–846 of *Pb*LAP3, has been removed from the *Pb*LAP3 coding sequence. Plasmids were linearised with *Hin*dIII and *Sac*II restriction enzymes to remove the vector backbone, and transfected into purified schizonts as previously described (Janse et al., 2006). Transgenic parasite lines were obtained by pyrimethamine selection followed by limiting dilution cloning as previously described (Janse et al., 2006). Genomic DNA extraction and Southern blot were performed as previously described (Dessens et al., 1999). All clonal transgenic parasite populations were checked for the absence of wildtype parasites by diagnostic PCR with primers pDNR-LAP3-F (5′ACGAAGTTATCAGTCGAGGTACCTAGCGGAAACAACAATGTTC3′) and LAP3–3′R (5′CCTCAAGATAGTTACGAATTTAAC3′).

### 2.4 Western blot

Parasite samples were heated directly in SDS–PAGE loading buffer at 70 °C for 10 min. Proteins were fractionated by electrophoresis through NuPage 4–12% Bis-Tris precast gels (Invitrogen) and transferred to polyvinylidene fluoride (PVDF) membrane according to the manufacturer’s instructions. Membranes were blocked for non-specific binding in PBS supplemented with 0.1% Tween 20 and 5% skim milk for 1 h at room temperature. Goat polyclonal antibody to GFP conjugated to horse radish peroxidase (HRP) (ab6663; Abcam, United Kingdom) diluted 1:5000 was applied to the membrane for 1 h at room temperature. After washing, signal was detected by chemiluminescence (ECL western blotting substrate, Pierce, United Kingdom) according to the manufacturer’s instructions.

### 2.5 Microscopy

For assessment of fluorescence, live parasite samples were assessed, and images captured, on a Zeiss LSM510 confocal microscope. ER-ID Red (Enzo Life Sciences, United Kingdom) was used to stain the ER according to the manufacturer’s instructions. Parasites were prepared for electron microscopy by overnight fixation in 2.5% glutaraldehyde/2.5% paraformaldehyde/0.1 M Na cacodylate buffer at 4 °C. Samples were post-fixed with 1% osmium tetroxide/0.1 M Na cacodylate buffer, washed with buffer followed by MilliQ water, en bloc stained with 3% aqueous uranyl acetate, dehydrated in ascending ethanol concentrations, rinsed briefly in propylene oxide, then embedded and polymerised in Taab epoxy resin. Ultrathin sections were cut and mounted on Pioloform-coated copper grids and stained with lead citrate. Immunogold labelling was carried out as previously described (McDonald et al., 1995) using rabbit polyclonal antibody to GFP (ab6556; Abcam) diluted 1:500 and goat-anti-rabbit IgG 10 nm gold-conjugated (BB International, United Kingdom) diluted 1:400. Samples were examined on a Jeol 1200EX Mark II transmission electron microscope and digital images recorded with a 1 K 1.3 M pixel High Sensitivity AMT Advantage ER-150 CCD camera system.

## 3 Results

### 3.1 Crystalloid formation occurs during early ookinete development

**Fig. 1:**
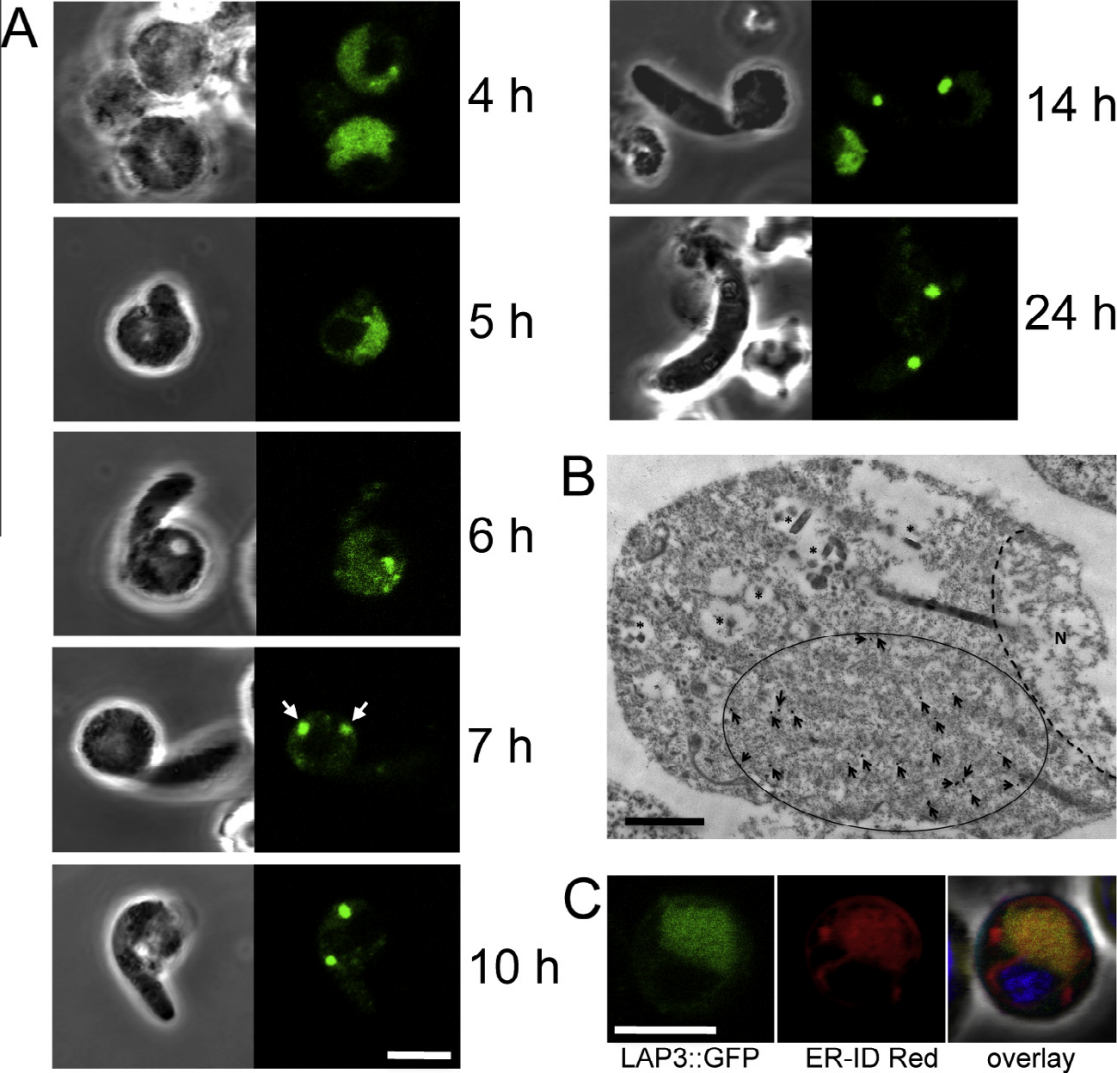
Crystalloid formation during *Plasmodium berghei* ookinete development in parasite line *Pb*LAP3/GFP. (A) Confocal microscope images showing typical subcellular distribution of LAP3::GFP at different time points after gametogenesis. White arrows at 7 h mark early crystalloids. Scale bar = 5 μm. (B) Immuno electron micrograph of a *Pb*LAP3/GFP gametocyte section labelled with anti-GFP primary antibodies and gold-conjugated secondary antibodies. The presence of gold particles (marked by arrows) is limited to a large extranuclear region of cytoplasm (encircled). Also marked are the nucleus (N) and hemozoin-containing vacuoles (*). Scale bar = 1 μm. (C) Confocal microscope image of a zygote at 5 h post-gametogenesis, co-stained with the endoplasmic reticulum marker ER-ID Red. Hoechst DNA stain (blue) labels the nucleus in the overlay image. Scale bar = 5 μm.

We previously described parasite line *Pb*LAP3/GFP, which expresses *Pb*LAP3::GFP fusion protein that is efficiently targeted to the crystalloid (Saeed et al., 2010). This parasite line therefore provides a useful molecular marker for the crystalloid organelle, which we used here to study its formation during ookinete development. Ookinete cultures were set up from gametocytemic mouse blood and crystalloid formation was assessed at different times post-gametogenesis. The first clear signs of ookinete development were visible at 5 h, with the spherical zygotes displaying a short protrusion corresponding to the apical end of the ookinete (Fig. 1A). The distribution of GFP fluorescence at 5 h was similar to earlier time points including female gametocytes, corresponding to a large and somewhat patchy extranuclear region (Fig. 1A). Consistent with this, immunogold electron microscopy (IEM) of *Pb*LAP3/GFP gametocytes showed labelling of a large and seemingly discrete region of extranuclear cytoplasm (Fig. 1B). Although the relatively harsh fixation protocol required for optimal antibody-antigen binding in IEM poorly preserves the subcellular structures, precluding a definitive allocation of the label, its distribution is consistent with that of the extensive ER present in female *P. berghei* gametocytes (Olivieri et al., 2015). In addition, LAP3::GFP co-localised with a red fluorescent ER marker in live cells (Fig. 1C). These combined observations indicate that LAP3 is present predominantly in the ER lumen in female gametocytes and during the early stages of ookinete development, which is in full agreement with the presence of a canonical ER signal peptide in *Pb*LAP3 and its orthologues (Claudianos et al., 2002; Pradel et al., 2004). At 6 h the distribution of LAP3::GFP had become more punctate, possibly reflecting accumulation of the protein around ER exit sites (Fig. 1A). The first clear signs of crystalloid formation became apparent by 7 h: retorts were showing one or two evident, albeit weak, fluorescent spots (Fig. 1A). By 10 h crystalloid formation was all but complete, the cells possessing two bright fluorescent spots within the spherical part of the retort, and 4 h later the crystalloids had begun moving into the ‘ookinete’ part of the retort (Fig. 1A). The crystalloids remained until ookinete development had completed, after which they were found located mostly, but not exclusively, at opposite sides of the nucleus (Fig. 1A). The large majority of mature ookinetes at 24 h post-gametogenesis possessed two crystalloids (77%), with the remainder having either one (5%) or three (18%) crystalloids (*n* = 100). The combined observations demonstrate that crystalloid formation takes place predominantly in the spherical ‘zygote’ part of the retort during the first 10 h of ookinete development.

### 3.2 Crystalloid biogenesis involves transport and assembly of subunit vesicles

**Fig. 2:**
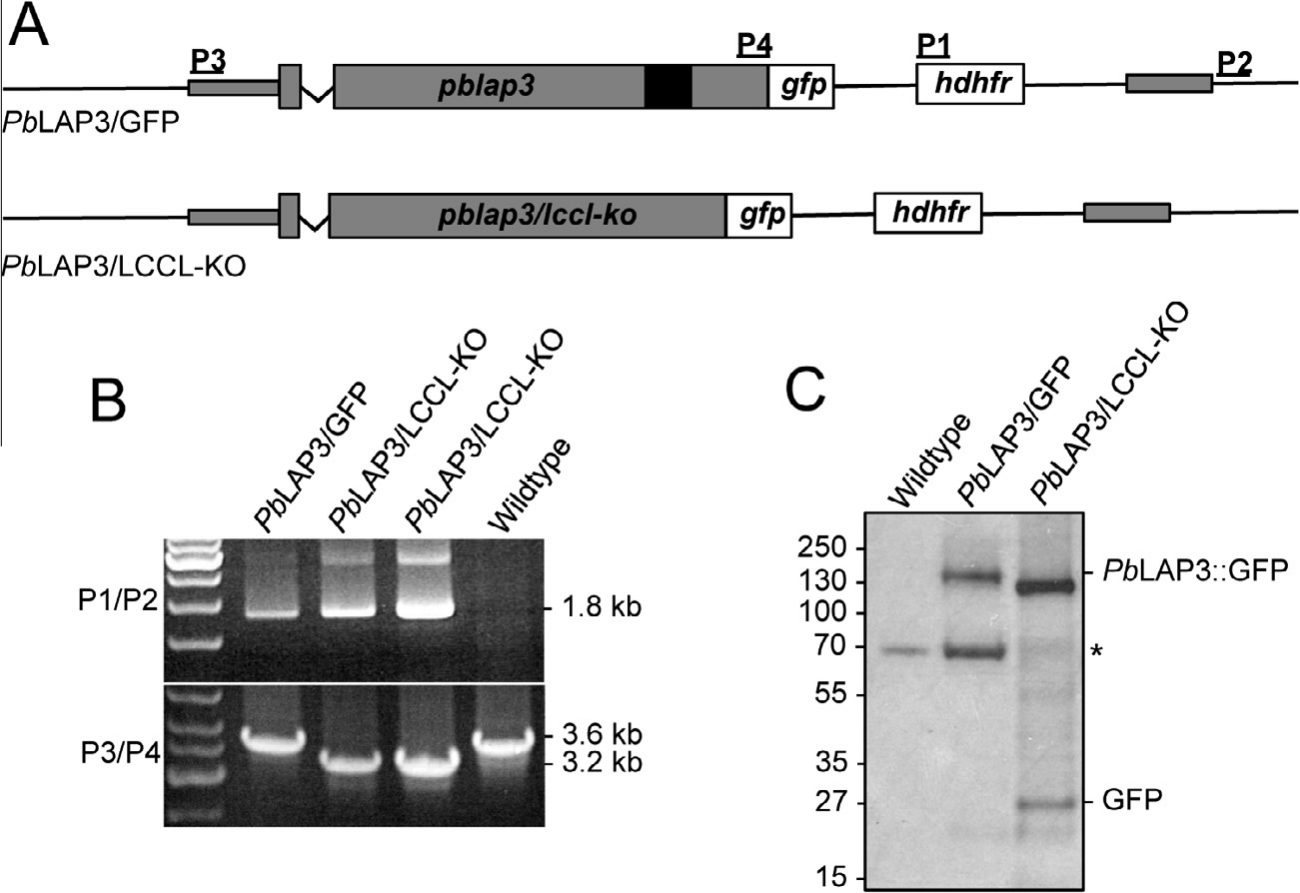
Molecular analyses of *Plasmodium berghei* parasite line *Pb*LAP3/LCCL-KO parasites. (A) Schematic diagram of the *pblap3* allele structure in parasite lines *Pb*LAP3/GFP and *Pb*LAP3/LCCL-KO. Indicated are the primer sites (P1 - P4) used for diagnostic PCR. The LCCL domain is denoted with a black box. (B) Diagnostic PCR with primers P1 (5′ACAAAGAATTCATGGTTGGTTCGCTAAACT3′) and P2 (5′CCTCAAGATAGTTACGAATTTAAC3′) for integration of the human dihydrofolate reductase (*hdhfr*) selectable marker gene into the *pblap3* locus (P1/P2), and with primers P3 (5′ACGAAGTTATCAGTCGAGGTACCTAGCGGAAACAACAATGTTC3′) and P4 (5′ATGAGGGCCCCTAAGCTATTTTTAATAATTTGTATCGAAAGTATAGTTG3′) for absence/presence of the LCCL domain deletion (P3/P4). (C) Western blot of gametocytes using anti-GFP antibodies. The blot shows bands corresponding to the full-length (∼150 kDa) and LCCL domain-lacking (∼135 kDa) LAP3::GFP fusion proteins, cleaved GFP (∼27 kDa), and a ∼65 kDa host cell protein (*) that cross-reacts with the antibody (Saeed et al., 2012; Tremp et al., 2013). Molecular weight markers are indicated on the left hand side.

All LAP family members possess at least one LCCL domain, with the exception of LAP5. The latter is included in the family by virtue of being a close structural paralogue of LAP3, with an identical domain topology except for the (missing) LCCL domain (Dessens et al., 2011). The fact that *Pb*LAP5 is necessary for normal parasite development and sporozoite transmission in its own right (Ecker et al., 2008) suggested that the LCCL domain of *Pb*LAP3 could be non-essential for protein function. To test this hypothesis the LCCL domain was removed from *Pb*LAP3, thereby turning it into a *Pb*LAP5-like protein. To achieve this, the sequence corresponding to the LCCL domain was removed from the *pblap3::gfp* allele to generate parasite line *Pb*LAP3/LCCL-KO (Fig. 2A). This parasite expresses *Pb*LAP3 without its LCCL domain, but with a C-terminal GFP tag. Different clonal populations of this parasite line were obtained and validated by diagnostic PCR, which showed integration of the selectable marker gene into the *pblap3* locus, as well as the presence of the ∼400 bp deletion in the mutant *lap3::gfp* allele (Fig. 2B). Gametocytes of *Pb*LAP3/LCCL-KO parasites exhibited GFP fluorescence in gametocytes similar to *Pb*LAP3/GFP parasites, and readily developed into ookinetes in culture. Western blot with anti-GFP antibody detected a GFP fusion protein in *Pb*LAP3/LCCL-KO parasites that was ∼15 kDa smaller than the equivalent LAP3::GFP fusion protein detected in *Pb*LAP3/GFP parasites, consistent with deletion of the LCCL domain (Fig. 2C). In addition, an approximately 27 kDa protein likely corresponding to cleaved GFP was present in the *Pb*LAP3/LCCL-KO parasite line. The enhanced cleavage of GFP in this parasite compared with *Pb*LAP3/GFP could reflect an altered conformation of the LAP complex in response to the LCCL deletion of *Pb*LAP3.

**Fig. 3:**
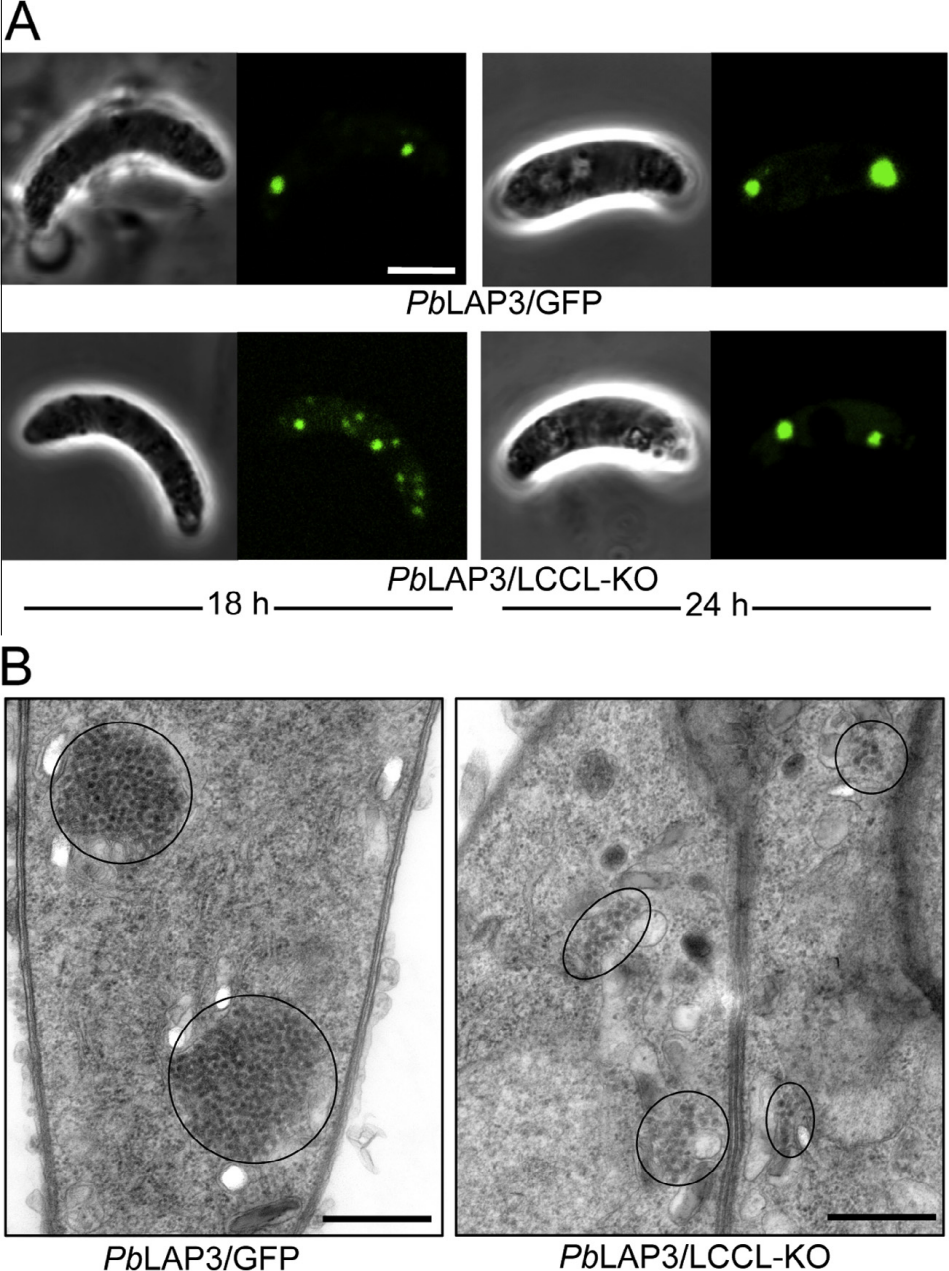
Phenotypic analyses of *Plasmodium berghei* parasite line *Pb*LAP3/LCCL-KO ookinetes. (A) Confocal microscope images of cultured *Pb*LAP3/LCCL-KO ookinetes at 18 h and 24 h post-gametogenesis, compared with *Pb*LAP3/GFP control ookinetes. Scale bar = 5 μm. (B) TEM images of *Pb*LAP3/GFP and *Pb*LAP3/LCCL-KO ookinetes at 18 h post-gametogenesis. Crystalloids are encircled. Scale bar = 500 nm.

**Fig. 4:**
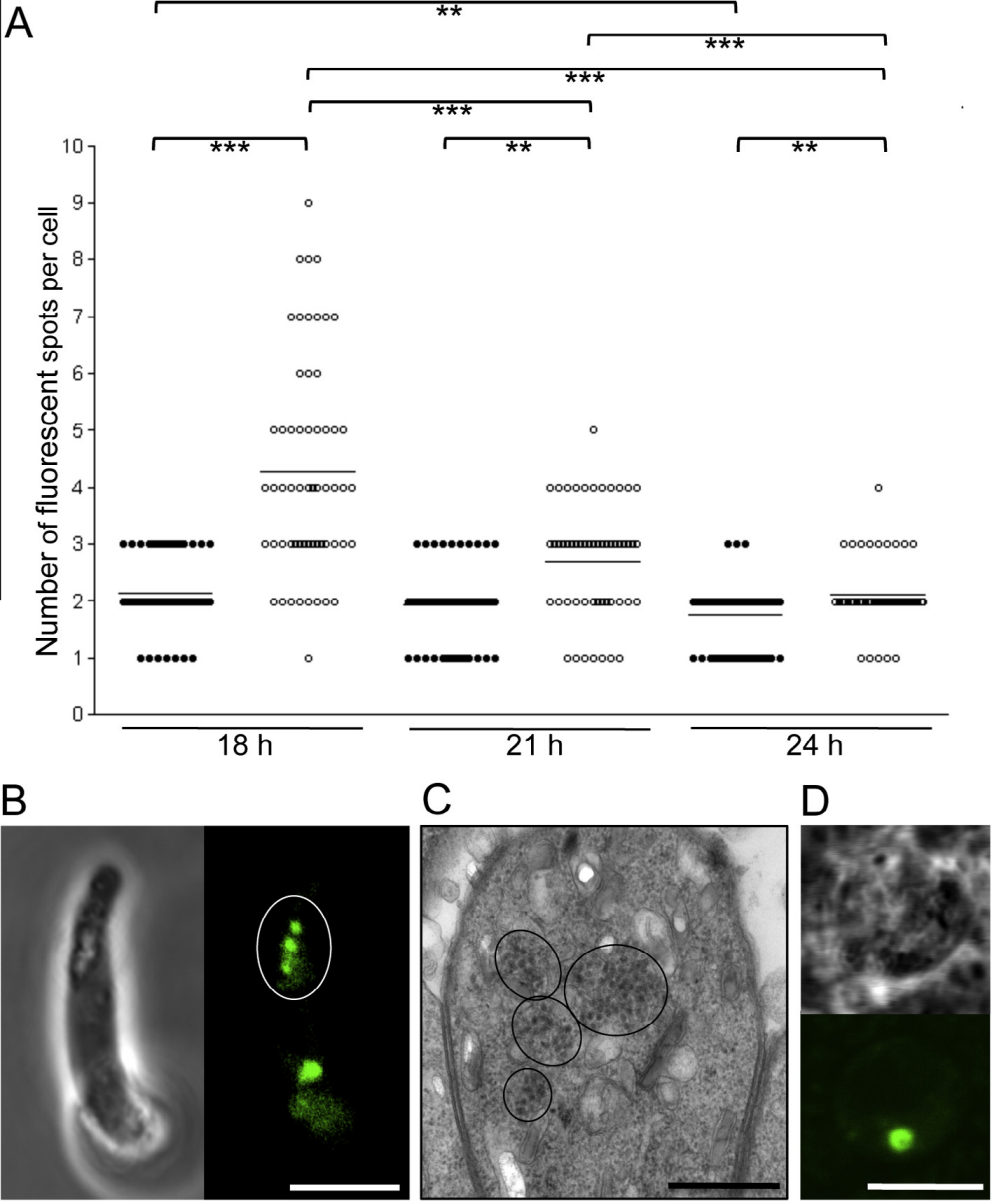
Crystalloids form via an assembly process. (A) Time course of the number of fluorescent spots/crystalloids per cell in cultured *Plasmodium berghei* parasite line *Pb*LAP3/LCCL-KO ookinetes (open circles) at 18, 21 and 24 h post-gametogenesis, compared with parasite line *Pb*LAP3/GFP ookinetes (closed circles). Horizontal lines denote mean values. Statistically significant differences are indicated: ^∗∗^*P < *0.001 and ^∗∗∗^*P < *0.0001 (Mann–Whitney). (B) Confocal microscope image (scale bar = 5 μm) and (C) transmission electron microscope image (scale bar = 500 nm) of ‘merging’ crystalloids in *Pb*LAP3/LCCL-KO ookinetes. Crystalloids are encircled. (D) Confocal microscope image of a spherical young *Pb*LAP3/LCCL-KO oocyst located on an *Anopheles stephensi* midgut, possessing a single large crystalloid. Scale bar = 5 μm.

Cultured ookinetes examined by confocal microscopy 24 h post-gametogenesis displayed no apparent differences between *Pb*LAP3/LCCL-KO and *Pb*LAP3/GFP control parasite lines, the majority of ookinetes displaying two fluorescent spots characteristic of the crystalloids (Fig. 3A). Indeed, both parasite lines had comparable infectivity in mosquitoes (58 ± 22 oocysts per mosquito for *Pb*LAP3/GFP; 35 ± 9 for *Pb*LAP3/LCCL-KO, *n* = 20; *P* = 0.98, Mann–Whitney test) and formed sporozoites that were readily transmitted by mosquito bite. These results demonstrate that *Pb*LAP3 without its LCCL domain retains biological activity. In contrast, when *Pb*LAP3/LCCL-KO ookinetes were examined at 18 h post-gametogenesis they looked markedly different from *Pb*LAP3/GFP control ookinetes, possessing notably more and generally smaller fluorescent spots (Fig. 3A). The same was observed in different clones of the LCCL domain deletion mutant, indicating this phenotype was not the result of clonal variation. TEM examination of these ookinetes revealed the presence of more and much smaller clusters of subunit vesicles (Fig. 3B). Assessing the number of fluorescent spots/crystalloids in a time course showed a gradual decrease in their number (Fig. 4A), indicating that the mini-crystalloids congregate during crystalloid formation. On many occasions we observed *Pb*LAP3/LCCL-KO ookinetes with several smaller crystalloids in close proximity of each other, seemingly in the process of merging (Fig. 4B). A similar process was observed by TEM (Fig. 4C). Interestingly, in control LAP3/GFP ookinetes there was also a significant, albeit small, decrease in the mean number of crystalloids per cell between 18 and 24 h post-gametogenesis (Fig. 4A), indicating that in wildtype ookinetes, also, crystalloids form by an assembly process. Indeed, when we examined young oocysts on the basal side of *A. stephensi* midguts at 2 days p.i., the large majority (96%, *n* = 50) possessed only a single large crystalloid (Fig. 4D), with the remaining oocysts possessing two closely apposed crystalloids. Thus, crystalloid assembly continues up to development of young oocysts.

### 3.3 Crystalloid assembly requires microtubule-based vesicle transport

**Fig. 5:**
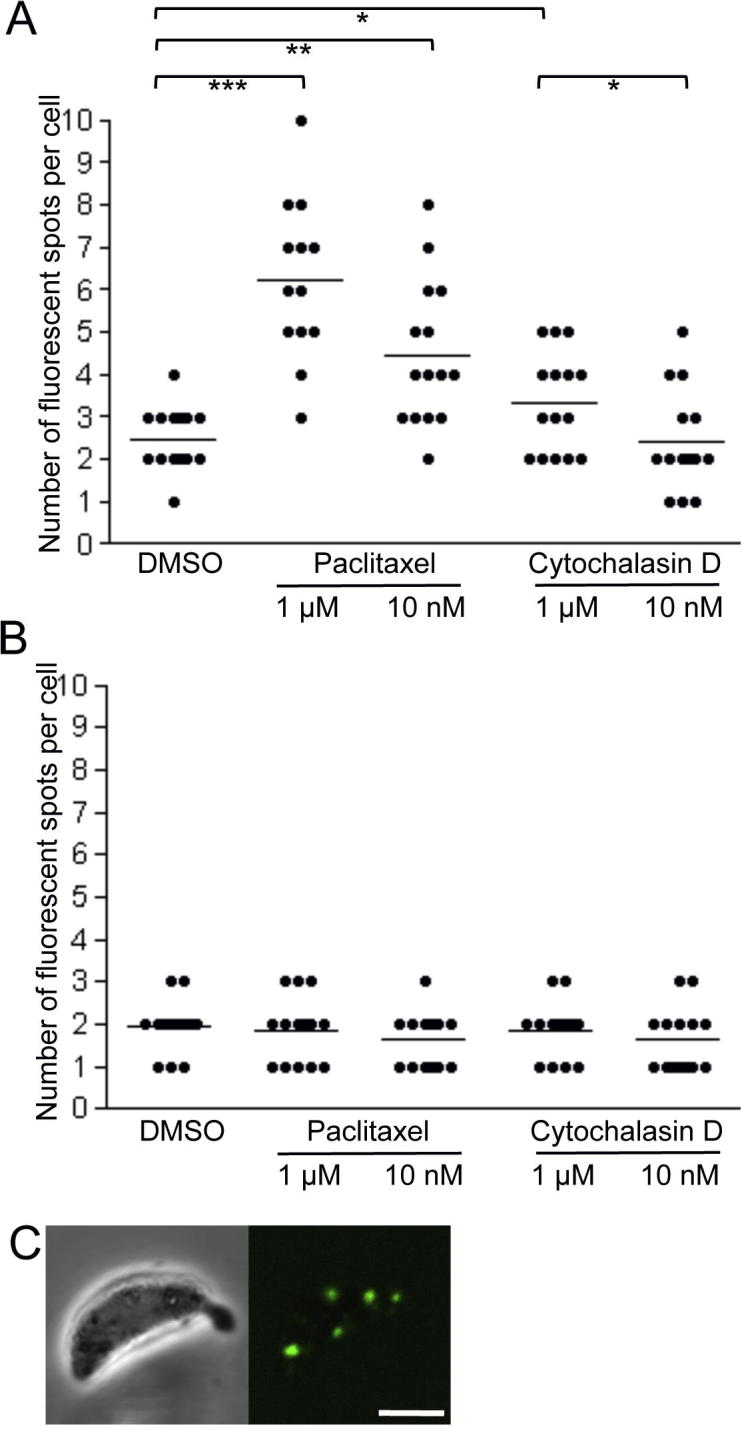
Inhibitors of vesicle transport affect crystalloid biogenesis. (A) Number of fluorescent spots per cell in cultured *Plasmodium berghei* parasite line *Pb*LAP3/LCCL-KO ookinetes and (B) *Pb*LAP3/GFP ookinetes at 24 h post-gametogenesis in the presence of paclitaxel or cytochalasin D and compared with DMSO solvent controls. Inhibitors were added at 18 h post-gametogenesis. Horizontal lines denote mean values. Statistically significant differences are indicated: ^∗^*P < *0.05, ^∗∗^*P < *0.001 and ^∗∗∗^*P < *0.0001 (Mann–Whitney). (C) Confocal microscope image of a *Pb*LAP3/GFP ookinete at 24 h post-gametogenesis with 1 μM paclitaxel added at 6 h post-gametogenesis, showing multiple crystalloids. Scale bar = 5 μm.

The apparent transport and assembly of crystalloid subunits suggested that crystalloid formation requires vesicle transport. There is extensive evidence that transport of membrane vesicles in eukaryotic cells takes place along tracks of cytoskeletal polymers (Goodson et al., 1997). To investigate this hypothesis, we tested the effects of chemical inhibitors of cytoskeleton-based cargo transport. In a first set of experiments, inhibitors were added to *Pb*LAP3/LCCL-KO ookinete cultures at 18 h and the effects on crystalloid assembly were assessed at 24 h. Paclitaxel, which interferes with microtubule dynamics and impedes microtubule-based cargo transport in vivo (Hamm-Alvarez et al., 1994; Sonee et al., 1998; Schnaeker et al., 2004; Hellal et al., 2011) had a marked effect on crystalloid formation in a dose-dependent manner, compared with the DMSO solvent control that did not affect crystalloid assembly (Fig. 5A). Paclitaxel at 1 μM effectively stopped progression of crystalloid assembly, resulting in ookinetes with more and smaller spots similar to the 18 h starting point. To a lesser extent, cytochalasin D, which interferes with actin filament formation and impedes actin/myosin-based cargo transport, significantly inhibited this process (Fig. 5A). In contrast, there was no discernible effect of either of the inhibitors on crystalloid formation in control *Pb*LAP3/GFP ookinetes (Fig. 5B). This was as expected, because assembled crystalloids were already present at 18 h when the inhibitors were added (Fig. 3A). These observations indicate that crystalloid biogenesis requires both microtubule- and actin filament-dependent cargo transport.

**Fig. 6:**
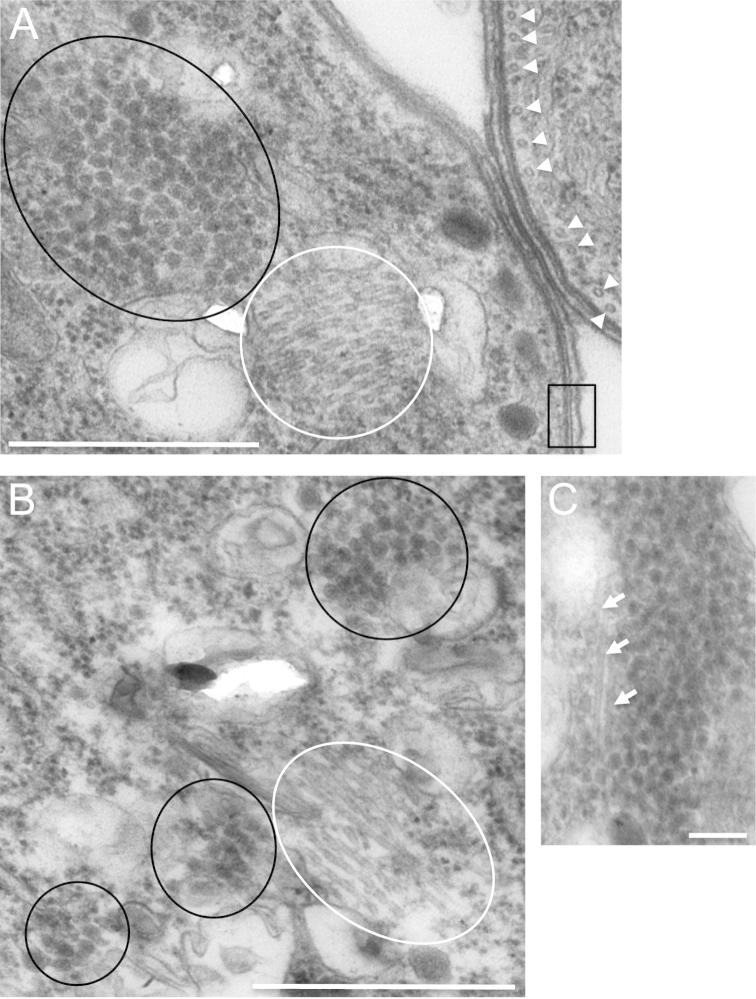
Association of microtubules and crystalloids. Transmission electron microscopy images of thin sections of paclitaxel-treated *Plasmodium berghei* parasite line *Pb*LAP3/GFP ookinetes. (A) Transverse section through a bundle of microtubules (encircled white) adjacent to a crystalloid (encircled black). White arrowheads mark subpellicular microtubules in a neighbouring ookinete. Black box marks pellicle membranes. Scale bar = 500 nm. (B) Slightly more longitudinal cross-section through a bundle of microtubules (encircled white) within a crystalloid assembly site (crystalloids encircled black). Scale bar = 500 nm. (C) Microtubules (white arrows) embedded within a crystalloid. Scale bar = 100 nm.

To test the effects of cargo transport inhibitors on crystalloid formation in wildtype parasites, 1 μM paclitaxel was added 6 h post-gametogenesis to *Pb*LAP3/GFP ookinete cultures. This is the earliest time this compound can be added without preventing development of mature ookinetes (Kumar et al., 1985). At 24 h post-gametogenesis, paclitaxel-treated *Pb*LAP3/GFP ookinetes possessed significantly more and smaller spots (Fig. 5C) than the DMSO-treated controls (paclitaxel: 2–8 spots, mean 4.5; DMSO: 1–3 spots, mean 1.6; *n* = 20; *P* < 0.01, Mann–Whitney test). Control and paclitaxel-treated ookinetes had comparable viability levels 24 h post-gametogenesis (DMSO 98% viability; paclitaxel 97% viability; *n* = 100), indicating that the increase in the number of fluorescent spots was not the result of cytotoxicity of the inhibitor to the parasite. Moreover, TEM examination of paclitaxel-treated LAP3/GFP ookinetes showed an overall normal development of subcellular organelles and structures, including the subpellicular microtubules (Fig. 6). Interestingly, bundles of microtubules were observed in close proximity to crystalloids/crystalloid assembly sites (Fig. 6). Similar structures were not found in untreated ookinetes. These combined results indicate that crystalloid formation involves microtubules. The fact that we can replicate, at least in part, the *Pb*LAP3/LCCL-KO phenotype in *Pb*LAP3/GFP parasites by adding cargo transport inhibitors suggests that the basic processes of crystalloid biogenesis are the same between the wildtype and mutant parasites. Accordingly, the *Pb*LAP3/LCCL-KO mutant parasite appears to exhibit attenuated crystalloid genesis manifested in a delay in crystalloid assembly. Despite this delay, normal crystalloids are present by the time of ookinete-to-oocyst transition (Fig. 3A).

### 3.4 Knockout of PbLAP3 abolishes crystalloid biogenesis

**Fig. 7:**
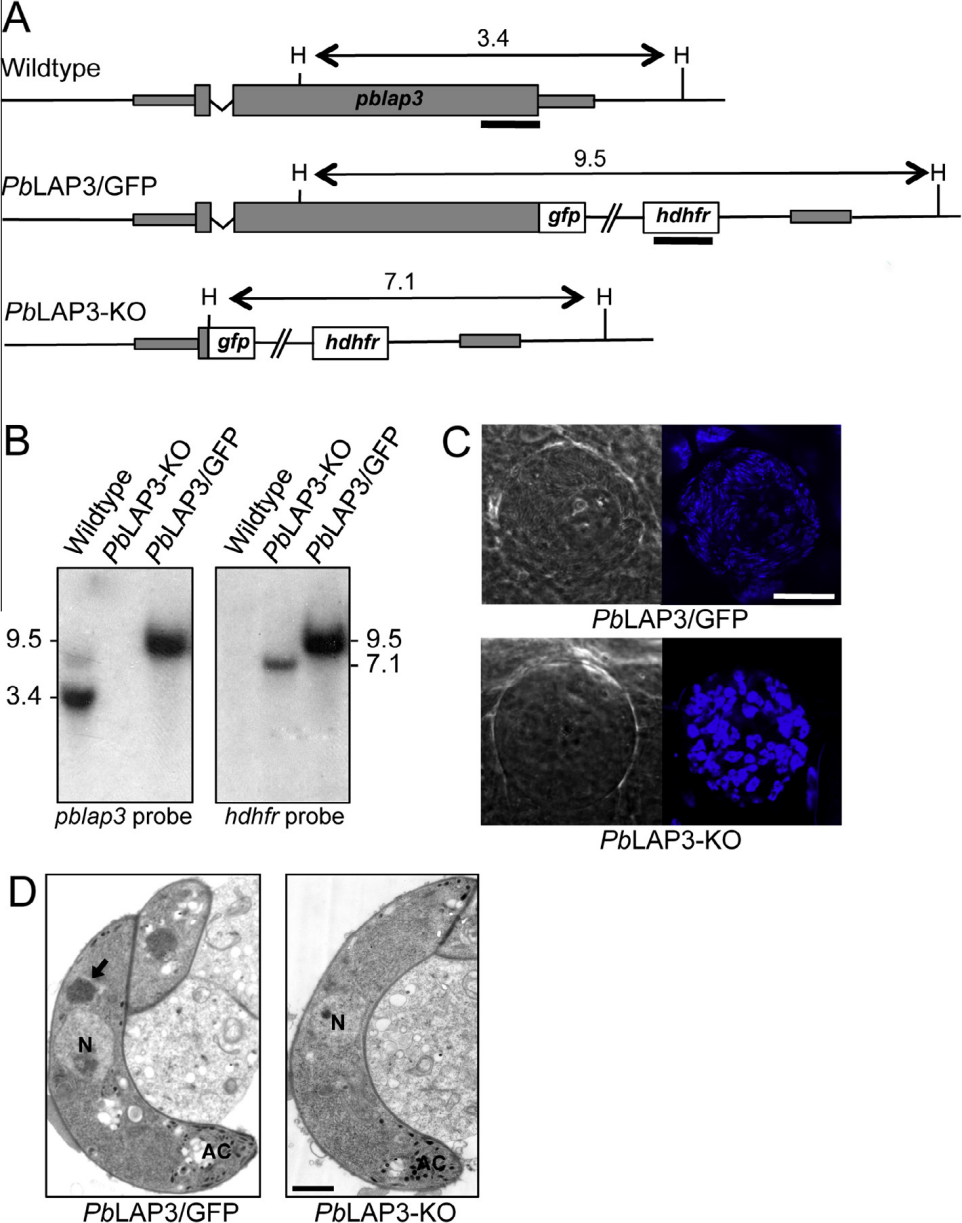
Genotypic and phenotypic analyses of *Plasmodium berghei* LAP3 null mutant parasites. (A) Schematic diagram of the *pblap3* allele structure in parental wildtype and transgenic *Pb*LAP3/GFP and *Pb*LAP3-KO parasite lines. Indicated are the human dihydrofolate reductase (*hdhfr*) selectable marker gene, the *pblap3* locus, *Hin*dIII restriction sites (H), sizes of the predicted *Hin*dIII restriction fragments, and regions used as probes (thick black lines). (B) Southern blot analysis of *Hin*dIII-digested genomic DNA. Indicated are the sizes of bands in kb. (C) Confocal microscope images of a typical sporulating (*Pb*LAP3/GFP) and non-sporulating (*Pb*LAP3-KO) oocyst at 2 weeks p.i. Hoechst DNA stain (blue) labels the nuclei. Scale bar = 10 μm. (D) Transmission electron micrographs of mature ookinete sections typical of *Pb*LAP3/GFP and *Pb*LAP3-KO parasite lines. The crystalloid is marked with a black arrow. Also indicated are the nucleus (N) and apical complex (AC). Scale bar = 1 μm.

To determine if the delayed crystalloid biogenesis observed in the *Pb*LAP3/LCCL-KO parasites was a complete or partial loss-of-function phenotype, we generated a *Pb*LAP3 null mutant parasite line named *Pb*LAP3-KO using double crossover homologous recombination (Fig. 7A). Correct integration of the selectable marker into the target locus was confirmed by Southern analysis of *Hin*dIII-digested genomic DNA (Fig. 7B): a *pblap3*-specific probe detected bands of 3.4 kb and 9.5 kb in wildtype and *Pb*LAP3/GFP parasites, respectively, but no signal in *Pb*LAP3-KO parasites, as expected (Fig. 7A and B). Conversely, a *hdhfr*-specific probe detected bands of 7.1 kb and 9.5 kb in *Pb*LAP3-KO and *Pb*LAP3/GFP parasites, respectively, but no signal in wildtype parasites, as predicted (Fig. 7A and B). *Pb*LAP3-KO parasites displayed normal blood stage development, produced gametocytes and readily formed oocysts in *A. stephensi* vector mosquitoes (58 ± 22 oocysts per mosquito for *Pb*LAP3/GFP; 56 ± 26 for *Pb*LAP3-KO, *n* = 20). However, the large majority of oocysts (∼98%) failed to produce sporozoites (Fig. 7C). In line with this observation, we were repeatedly unable to transmit this parasite by mosquito bite. The same phenotype was observed with a different clone of the *Pb*LAP3-KO line. By contrast, *Pb*LAP3/GFP control parasites exhibited normal sporulation (Fig. 7C) and were readily transmitted. These observations demonstrate that *Pb*LAP3 is necessary for the production of infective sporozoites in mosquitoes.

When we examined *Pb*LAP3-KO ookinetes by TEM we could not find any evidence of crystalloid biogenesis, while other known ookinete structures and organelles were normally present (Fig. 7D). Thin sections of control *Pb*LAP3/GFP ookinetes had crystalloids in 83% of distinct cells examined (*n* = 82), while none were found in equivalent sections of *Pb*LAP3-KO ookinetes (*n* = 71), demonstrating that *Pb*LAP3 is essential for crystalloid biogenesis (*P* < 0.0001, Fisher’s exact test). This observation strongly points to a functional link between crystalloid formation in the ookinete, and sporogenesis in the oocyst. The *Pb*LAP3-KO phenotype clearly is more severe than that of the *Pb*LAP3/LCCL-KO mutant, confirming that the latter is indeed an intermediate phenotype.

## 4 Discussion

**Fig. 8:**
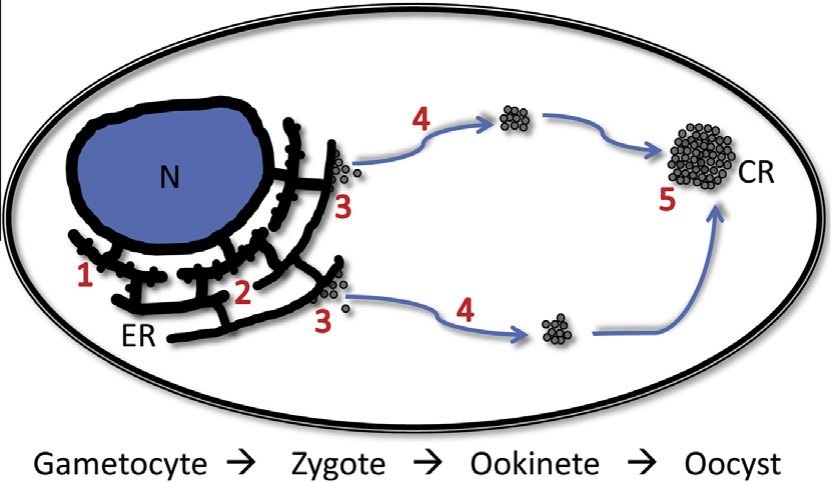
Proposed model of crystalloid biogenesis in *Plasmodium berghei*. A single cell is depicted, which represents the transformation from gametocyte, via the zygote and ookinete, to oocyst. N, nucleus; ER, endoplasmic reticulum; CR, crystalloid. Key steps are indicated by numbers. Step 1: translation of the LCCL-lectin adhesive-like proteins (LAPs) in rough ER and translocation into ER lumen; Step 2: assembly of the LAP family members into a functional protein complex; Step 3: formation of the subunit vesicles at ER exit sites; Step 4: transport of the subunit vesicles to (typically two) assembly sites; Step 5: final merging of sub crystalloids into a single organelle in the oocyst.

This study shows that crystalloid biogenesis in the rodent malaria parasite species *P. berghei* is achieved via a process of sequential subunit vesicle formation, transport and coordinated assembly (Fig. 8), and that these processes are microtubule-dependent. These processes are likely to be conserved in human malaria parasite species such as *P. falciparum*, which possesses crystalloids virtually indistinguishable from those found in *P. berghei* (Meis and Ponnudurai, 1987). Our data show furthermore that crystalloid formation happens to a large extent during the early stages of ookinete development (Fig. 1A), but does not complete until oocyst transition, ultimately giving rise to a single crystalloid organelle in the oocyst (Fig. 4D).

The demonstrated localisation of the LAPs in the crystalloid (Carter et al., 2008; Saeed et al., 2010, 2013) suggests that the LAP complex is part of the cargo of its subunit vesicles. Interactions of major cargo molecules with the COPII machinery contribute to the formation of vesicles budding from the ER (Aridor et al., 1999). This could explain why deletion or alteration of *Pb*LAP3 adversely affects crystalloid formation, as such interactions could be compromised. In the *Pb*LAP3 null mutant we found no evidence of crystalloid assembly, indicating that the subunit vesicles are not formed in the first place. The LAPs are co-dependent for conformation and stability (Pradel et al., 2006; Simon et al., 2009; Saeed et al., 2012), and it is therefore probable that in the *Pb*LAP3 null mutant a functional LAP complex is unable to form in the ER lumen (step 2 in Fig. 8), which in turn could prevent formation of crystalloid subunit vesicles at their ER exit sites. This notion is further supported by observations that dysfunctional *Pb*LAP1 lacking its two tandem scavenger receptor cysteine-rich (SRCR) domains remains in the ER (Carter et al., 2008). By contrast, in the *Pb*LAP3/LCCL-KO mutant, subunit vesicles are clearly formed and engage with the intrinsic mechanisms of vesicle transport, allowing crystalloid assembly to proceed and produce normal crystalloids by the time of oocyst transition. In this mutant, subunit vesicle formation could be slowed down as a result of a suboptimal interaction of the altered LAP complex with the vesicle budding machinery (step 3 in Fig. 8).

Our observation that crystalloid biogenesis is sensitive to inhibitors of both microtubule- and actin filament-based transport (Fig. 5A) implies that a degree of filament switching takes place (Langford, 1995; Schroeder et al., 2010). The classic dual filament model of cargo transport uses microtubules for ‘long distance’ and actin filaments for local dynamic interactions (Schroeder et al., 2010). The same may be true for crystalloid formation, as the effect of cytochalasin D on vesicle assembly is much less pronounced than that of paclitaxel (Fig. 5A). Moreover, cytochalasin D added at 1 μM to *Pb*LAP3/LCCL-KO ookinete cultures at 6 h post-gametogenesis did not significantly increase the adverse effect on crystalloid assembly (1–5 spots, mean 3.0) compared with its addition at 18 h (2–5 spots, mean 3.3), despite having more time to interfere with the process. These observations suggest that the actin filament-based transport could indeed be acting downstream of microtubule-dependent transport.

Our data using LAP3/GFP parasites show that crystalloids form early in ookinete development, within the spherical part of the retort (Fig. 1A). Because this part of the cell does not possess a pellicle or subpellicular microtubules, these unusually stable cortical microtubules (Cyrklaff et al., 2007) are unlikely to be involved in crystalloid biogenesis. In many regions of the cytoplasm microtubules are much more dynamic polymers that undergo continual assembly and disassembly (Waterman-Storer and Salmon, 1997; Jordan and Wilson, 2004), and our observations suggest that an alternative and more dynamic microtubule system could be involved in crystalloid assembly. In the large majority of cells, crystalloid formation initially produces two ‘sub’ crystalloids (Fig. 1A), which persist in most ookinetes until oocyst transition, when they merge into a single crystalloid (Fig. 4D). This suggests that the vesicle assembly process that gives rise to crystalloid formation is not random but uses specific ‘assembly sites’. Given the microtubule dependence of crystalloid biogenesis, it is attractive to speculate that these assembly sites are orchestrated by microtubule organising centres (MTOCs), allowing subunit vesicles to move toward them along microtubules using dynein motors. This hypothesis is supported by our observation of microtubules in close proximity to crystalloids in paclitaxel-treated ookinetes (Fig. 6). Potential MTOCs in the zygote could include the spindle pole plaques (Sinden et al., 1985), cytoplasmic centrioles (Aikawa et al., 1984), or Golgi membranes that can nucleate microtubules (Miller et al., 2009; Zhu and Kaverina, 2013).

In the context of LAP family members forming a functional protein complex, it is not surprising that knockout of *Pb*LAP3 results in loss of sporozoite development and transmission, as is the case for its family members (Claudianos et al., 2002; Raine et al., 2007; Carter et al., 2008; Ecker et al., 2008; Lavazec et al., 2009). The fact that crystalloids are absent in the *Pb*LAP3 null mutant (Fig. 7D) shows that the *Pb*LAP3/LCCL-KO mutant exhibits a partial loss-of-function phenotype. An absence of crystalloid formation was also observed in *Pb*LAP1 (*Pb*SR) null mutants (Carter et al., 2008), and the lack of crystalloid formation reported here for *Pb*LAP3 thus makes it likely that this phenomenon is a shared feature of all LAP null mutants in *P. berghei*. The dramatic defect in sporozoite development in *Pb*LAP null mutants is thus consistent with an absence of crystalloid biogenesis, in turn suggesting that these organelles, or the cargo carried by them, are required for normal oocyst maturation and ensuing sporozoite transmission. Preventing crystalloid formation could therefore present an attractive strategy to block malaria transmission. One way to achieve this could be by chemically interfering with the formation of a functional LAP complex, effectively replicating the LAP null mutant phenotype. The gametocyte-specific expression of many LAPs (Pradel et al., 2006; Carter et al., 2008; Scholz et al., 2008; Simon et al., 2009; Saeed et al., 2010, 2013) means that LAP complex formation could be targeted in the human host, before the parasite enters the mosquito vector. As such, this transmission-blocking approach would not be reliant on the uptake of the inhibitor with the blood meal of the mosquito, which is required in transmission-blocking strategies that target development or progression of the life stages within the midgut lumen of the mosquito (i.e. gametes, zygotes and ookinetes). The ‘delayed death’ aspect of targeting LAP complex formation, and hence crystalloid biogenesis, would also benefit this strategy as the ookinete and oocyst loads in the mosquito are not reduced. The potential risk of increasing fitness of the insect by lowering its parasite burden is one of the caveats of current transmission-blocking strategies being developed (Dawes et al., 2009; Churcher et al., 2011), as reductions in sporozoite load could be counteracted by the mosquitoes being infective for longer, increasing their vectorial capacity. We therefore propose that transmission blockade through targeting the crystalloid organelle could provide a valuable new approach to complement the existing arsenal of malaria transmission control strategies being employed or developed.

The discoveries reported here regarding LAP function and crystalloid formation are also relevant in the context of other apicomplexan parasites. Biogenesis of crystalloids by active vesicle assembly could be a specific adaptation of the genus *Plasmodium*, since many other genera (including the medically and veterinary important *Toxoplasma*, *Eimeria*, *Babesia* and *Theileria*) do not possess crystalloids, but do encode LAP orthologues. A conserved function of apicomplexan LAPs in vesicle, rather than crystalloid, formation would allow for a role that could potentially serve the broad spectrum of life cycles present among members of this large and important phylum.
